# Cocaine in Ophthalmic Work

**Published:** 1893-10-14

**Authors:** J. Tatham Thompson

**Affiliations:** Ophthalmic Surgeon to the Cardiff Infirmary


### New Drugs and Preparations.

[We should be glad to receive, at our Office, 428, Strand, London, W.C., from the manufacturers, specimens of all new preparations and drugs which may be brought out from time to time.]

### COCAINE IN OPHTHALMIC WORK.

By J. Tatham Thompson, M.B., &c., Ophthalmic Surgeon to the Cardiff Infirmary.

Cocaine is best employed in ophthalmic surgery in solutions of various strengths. For all practical purposes three will suffice, viz., two, four, and ten per cent. solutions. The one found most useful for nearly all purposes is the four per cent. solution.

It need hardly be pointed out that great care should be exercised that the preparation should be at least aseptic, if not antiseptic, and a very useful method of preparing it in such a form as to be aseptic and likely to preserve to the fullest possible extent its asepticism and anæsthetic properties, is the following: The cocaine should be dissolved in freshly boiled distilled water, and in the weaker solutions an equal quantity of boracic acid added. In the stronger solution four or five per cent. of boracic acid is quite sufficient.

Cocaine undoubtedly loses a certain amount of activity by long keeping in solution, and in critical and delicate operations it is of the utmost importance that the solution should be recently prepared and perfectly aseptic. It will be found, however, that with care duly exercised that no bottle shall remain uncovered and the drop tube carefully cleansed in an antiseptic after use, such preparation will keep perfectly well for considerable periods. If, however, the slightest sediment or cloudiness is apparent the preparation should be at once condemned.

Cocaine anæsthesia frequently proves of great use in enabling the practitioner to freely examine the eye in cases of extreme photophobia and blepharospasm, and here it is well to use the two per cent. solution. The stronger preparations seem temporarily to increase the spasm, but if some moments are allowed to elapse after instilling a few drops of the weak preparation, and the lids in the meantime are forcibly held sufficiently apart to prevent the expulsion of the drops, it will be found that the spasm is soon overcome by one or two subsequent instillations, and that a satisfactory examination can be made.

When it is desired that a rapid mydriatic effect shall be obtained, so as to get a good view of the media or fundus, a few drops of cocaine should be applied before using the homatropine or atropine. Whether or no there are any dilator muscle fibres in the iris is a disputed point; but it seems to be beyond dispute that after full paralysis of the sphincter iridis has been obtained, one may produce further dilatation of the pupil by the action of cocaine.

In anæsthetising to aid removal of foreign bodies from the conjunctival sacs or from the cornea, the four per cent. solution is the most useful. Here the effect is best got by rapid repeated applications of a small quantity. If the instillation is proceeded with too slowly, the mydriasis produced may be very bothering, as it is exceedingly hard to distinguish a small foreign body against the black background of a dilated pupil. Cocaine anæsthesia of the conjunctiva and cornea is an enormous help both to practitioner and patient, as a preliminary to the application of strong astringents and caustics. In the case of silver nitrate, it has a second use, as applied quickly after the caustic, it neutralizes any excess, forming the inert chloride of silver.

To allay the constant pain and photophobia in superficial injuries of the cornea, and in superficial keratitis, frequent applications of the weak solution will be found to answer better than the use of strong solutions at longer intervals. In the rare keratitis superficialis punctata, cocaine is contra-indicated, tending to increase the congestion. In iritis and irido-cyclitis, it is a great help to allay pain, and distinctly aids the use of atropine as a mydriatic, whilst it also seems to have a physiological action in reducing the congestion. Here the four per cent. solution acts best.

In the treatment of glaucoma it is often found that the application of eserine drops gives rise to great pain; this can be obviated by using cocaine either before or along with the eserine; the slight mydriatic properties of the former do not seem to have any perceptible effect in the presence of the powerful myotic eserine.

It is as an aid to operative procedure, however, that cocaine reaches the highest point in its value in ophthalmic practice. It has, indeed, worked a marvellous change in the ease and safety with which operations on the eye are carried out. One difficulty must be pointed out, however, and that is that when we are dealing with an eye in a highly-congested state, or with any increased intro-ocular tension. cocaine has not anything like the same effect as a local anæsthetic.

In the simple operation for removing tarsal cysts, the easiest way is to first anæsthetise the conjunctival surfaces by instillation of a few drops of four per cent. solution, and then introducing beneath the lid and over the cyst a little absorbent wool soaked in the cocaine; this should be left *in situ* for three or four minutes, at the end of which time it is usually a painless procedure to cut down on and scrape out the cyst. In cases of lacrymal stricture, if the obstruction is in the canaliculus the same method of application answers; but if the stricture is below the sac. it is well to inject a few drops of four per cent. solution into the sac; even then the anæsthesia is a very uncertain quantity.

For strabismus tenotomy the conjunctiva should be first anæsthetised, then the opening made into tenon's capsule, and a few drops injected by means of a fine-nozzled syringe into the opening. After the lapse of two or three minutes the tenotomy may be completed. In performing a "muscular advancement" it is usually necessary to apply cocaine at intervals as the operation proceeds and the parts are exposed. At one time I used to inject cocaine into the sub-conjunctival tissue as a preliminary, endeavouring to reach tenon's capsule; but finding that it was very uncertain whether one reached the capsule, and that the infiltration of the sub-conjunctival tissues rather hindered, I gave it up, and now only inject the capsule after cutting down on it.

The anæsthesia required for paracentesis of the anterior chamber is very quickly attained. Two or three applications of the four per cent. solution very soon producing the desired condition. In cases of hypopion, and where one has to cut outwards through a corneal abscess after the method of Saemesch, more difficulty is found, owing probably to the congested state, and a rather longer application is usually required. When a cautery is applied to a corneal ulcer or to the sloughing edges of a corneal wound, I have found that a very rapid and complete anæsthesia was got by depositing some powdered cocaine over the region to be cauterised, washing the part with warm boracic lotion immediately before using the cautery.

Where the iris is to be operated on it is advisable to have the four per cent. solution applied at frequent intervals for ten or fifteen minutes; if, in spite of that, the iris is found to be still sensitive, Mr. Anderson Critchett's method of anææsthetising the anterior chamber and iris may be adopted. It is, however, accompanied by greatly increased risk of septic mischief if the instruments and preparation are not beyond suspicion. After making the corneal incision a small curette or spatula is introduced into the anterior chamber, and the cocaine solution dropped into the grooved channel. The same observations apply to the use of cocaine in cataract extraction. It is very important, however, that two points be observed. Firstly, that the cocaine solution shall not be used too strong; if so it is apt to produce a cloudy condition of the cornea, so much so, indeed, as to interfere with one's view of the structures beneath. Secondly, the eye should not be kept exposed during the anæsthetising process, and during any prolonged operation the cornea should be moistened from time to time with warm boracic acid solution, as the corneal epithelium tends to desquamate.

In excision of the eyeball cocaine is sometimes used, but, I think, should only be employed in cases where a general anæsthetic would be accompanied by grave danger to life. Where it is found necessary to use it, the superficial parts should be first anæsthetised, the conjunctival incision completed, and cocaine injected as the deeper structures are reached.

In plastic operations on the eyelids, subcutaneous injection of ten per cent. solution acts very well, and so long as the total amount injected is under one grain there is practically no risk.

Cocaine is useful also as an addition to certain ointments to allay the pain and irritation which would otherwise be produced, as, for instance, with the oxides of mercury and eserine ointments.

Lastly, in the form of cocaine gelatine discs, these are, of course, very portable, but uncertain in their action. There is also, I think, risk of septic injection, as it is difficult to ensure their perfectly aseptic condition. The common method of applying them to the conjunctival or corneal surface by means of a brush is unsafe, as it is impossible to be sure of its absolute cleanliness, it is better to apply them with the moistened end of a silver probe, which can be subsequently boiled.

